# Transcriptomic Analysis of the Adaptation of *Listeria monocytogenes* to Lagoon and Soil Matrices Associated with a Piggery Environment: Comparison of Expression Profiles

**DOI:** 10.3389/fmicb.2017.01811

**Published:** 2017-09-26

**Authors:** Anne-Laure Vivant, Jeremy Desneux, Anne-Marie Pourcher, Pascal Piveteau

**Affiliations:** ^1^UR OPAALE, IRSTEA, Rennes, France; ^2^Université Bretagne Loire, Rennes, France; ^3^Agroécologie, AgroSup Dijon, Institut National de la Recherche Agronomique, Université Bourgogne Franche-Comté, Dijon, France

**Keywords:** *Listeria monocytogenes*, transcriptome, pig manure, RNAseq, lagoon effluent, soil, microcosms

## Abstract

Understanding how *Listeria monocytogenes*, the causative agent of listeriosis, adapts to the environment is crucial. Adaptation to new matrices requires regulation of gene expression. To determine how the pathogen adapts to lagoon effluent and soil, two matrices where *L. monocytogenes* has been isolated, we compared the transcriptomes of *L. monocytogenes* CIP 110868 20 min and 24 h after its transfer to effluent and soil extract. Results showed major variations in the transcriptome of *L. monocytogenes* in the lagoon effluent but only minor modifications in the soil. In both the lagoon effluent and in the soil, genes involved in mobility and chemotaxis and in the transport of carbohydrates were the most frequently represented in the set of genes with higher transcript levels, and genes with phage-related functions were the most represented in the set of genes with lower transcript levels. A modification of the cell envelop was only found in the lagoon environment. Finally, the differential analysis included a large proportion of regulators, regulons, and ncRNAs.

## Introduction

*Listeria monocytogenes*, the causative agent of listeriosis, can be found in farm environments (Nightingale et al., 2004; Vilar et al., 2007; Esteban et al., 2008; Boscher et al., 2012; Pourcher et al., 2015). Depending on the type of livestock waste (solid or liquid manure, litter) and management (static pile, composting, storage in a tank, aerobic or anaerobic digestion), *L. monocytogenes* may survive waste treatment. In previous studies on piggery effluents, we provided evidence for higher prevalence of *L. monocytogenes* in the liquid phase of manures treated aerobically (lagoon effluents) than in raw manures (Pourcher et al., 2015) and demonstrated that viable cells of *L. monocytogenes* entered the viable but non-culturable state within the first hours of contact with piggery lagoon effluents (Desneux et al., 2016). We also observed the presence of *L. monocytogenes* in soils located in the vicinity of the lagoons (Pourcher et al., 2015). *L. monocytogenes* can survive for more than a month in soil amended or not with livestock effluents (Jiang et al., 2004; Hutchison et al., 2005; Nicholson et al., 2005; Moynihan et al., 2015). Its persistence depends on intrinsic factors (physiological state, concentration), and on abiotic (physical and chemical properties, sunshine, precipitation, temperature) and biotic environmental factors (production of inhibitory molecules, competition for substrate, protozoan grazing). The manure application method also affects the survival of *L. monocytogenes* in the soil. Indeed, Hutchison et al. (2004) reported that incorporating livestock effluent increases the survival of manure-borne pathogens after spreading of the effluent. The presence of *L. monocytogenes* in livestock feces can therefore contaminate the soil when livestock effluents are spread and, following rainfall events, can contaminate surface waters through runoff and groundwater through infiltration. While rainfall appears to be a major factor in the dissemination of *L. monocytogenes* in water systems, temperature and the presence of livestock are also important (Lyautey et al., 2007; Wilkes et al., 2009; Cooley et al., 2014). For example, in Canada, Lyautey et al. (2007) analyzed 314 samples of surface water and observed that the number of samples positive for *L. monocytogenes* was higher in areas close to cattle farms. Similarly, among the 53 soil samples from 23 Burgundy sites analyzed by Locatelli et al. (2013a) (three cow pastures, eight cultivated fields, 11 meadows, and one forest), *L. monocytogenes* was isolated only in soils from pastures.

The ability to survive in these environments can be explained by the extensive regulatory repertoire identified in the genome of *L. monocytogenes* (Glaser et al., 2001), which represents 7.3% of the genome of *L. monocytogenes* EGD-e. In response to environmental cues including starvation (Herbert and Foster, 2001), changes in temperature (Liu et al., 2002; Cacace et al., 2010; Mattila et al., 2011), pH (Abram et al., 2008; Giotis et al., 2008; Chen et al., 2011), and osmotic variations (Duche et al., 2002; Bergholz et al., 2012), *L. monocytogenes* can adapt its physiology by regulating gene expression. This includes genes encoding cell surface proteins, secreted proteins, transporters, and proteins involved in motility and chemotaxis. In addition to the coding part of the genome, evidence suggests that non-coding RNAs (ncRNAs) are key regulators of the environmental adaptation of *L. monocytogenes* (Christiansen et al., 2006; Mandin et al., 2007; Nielsen et al., 2008, 2011; Mellin and Cossart, 2012).

Only a few authors have performed differential analysis at gene-level resolution in complex matrices. These include food-related conditions, food matrices (cabbage, milk, turkey; Palumbo et al., 2005; Liu and Ream, 2008; Bae et al., 2011), and disinfectants used in the food industry such as the benzalkonium chloride (BZT; Fox et al., 2011; Casey et al., 2014). To date, only one study (Piveteau et al., 2011), has described transcriptional modifications induced by the transfer of *L. monocytogenes* to an environmental matrix. Whatever the strain analyzed (EGD-e, 6179, F6854, F2365, or 10403) and the technology used (DNA microarray, RT-PCR, RNA-seq), a similar scenario was described with higher transcript levels of genes involved in the transport of carbohydrates (Bae et al., 2011; Piveteau et al., 2011; Casey et al., 2014), in the metabolism of amino acids (Liu and Ream, 2008; Piveteau et al., 2011) or in motility and in chemotaxis (Palumbo et al., 2005; Casey et al., 2014). Interestingly, while genes involved in the virulence were not differentially transcribed in food matrices or in presence of BZT (Liu and Ream, 2008; Casey et al., 2014), they had lower transcript levels in soil extracts (Piveteau et al., 2011), suggesting an effect of the matrix on the regulation of gene expression of *L. monocytogenes*.

Given the significant role of manure application in the dissemination of *L. monocytogenes* in farmland and the lack of data on its physiology in the agricultural environment, it is important to characterize the transcriptome of this pathogenic bacterium in both livestock effluents and soil. The main objective of this study was thus to identify the response of a strain isolated from manure (*L. monocytogenes* CIP 110868) 20 min and 24 h after being transferred to a lagoon effluent or a soil extract by differential analysis using RNAseq.

## Materials and methods

### Bacterial strain and culture media

*L. monocytogenes* CIP 110868 (serogroup IVb) was isolated from pig manure. The strain was grown at 30°C in a nutrient broth supplemented with 0.3% glucose (NBG). Three independent inocula were prepared by inoculating 100 ml of NBG to an O.D_600 nm_ of 0.04 and incubating overnight at 30°C to an O.D_600 nm_ of 0.4. The cultures were then centrifuged at 5,000 g for 5 min and the pellets were suspended in 0.8% NaCl solution. After a second washing step, the cells were suspended in saline to reach a final concentration of 5 × 10^9^ CFU/ml.

### Sampling and preparation of soil extract and lagoon effluent microcosms

Soil and lagoon effluent were collected in sterile flasks from a pig manure treatment processing unit located in Brittany (France). The treatment consists of pre-storage and centrifugation of the manure to remove phosphorus. The liquid phase, which is biologically treated to reduce the level of nitrogen, is then left to settle. The liquid fraction in the settling tank is sent to an open-air lagoon where it is stored for 9–12 months. The sample of silty topsoil (0–20 cm) was collected near the lagoon. The two matrices were stored at 4°C until analysis.

Soil extract was prepared according to Piveteau et al. (2011). Briefly, 500 g of soil were mixed in 750 mL of water and autoclaved at 130°C for 1 h. The soil suspension was centrifuged at 10,000 g for 20 min and the supernatant was filtered on Whatman® 3 MM paper. The soil extract obtained after filtration was used after autoclaving at 120°C for 20 min.

The lagoon effluent extract was prepared by centrifugation of the effluent at 10,000 g for 10 min. The supernatant was filtered through Whatman® 3 MM paper and Whatman® qualitative filter paper Grade 2 (8 μm). The extract was then filter-sterilized on cellulose ester membrane pore size 3, 1.2, 0.8, 0.45, and 0.22 μm and transferred to sterile flasks. Sterility was confirmed by measuring culturable bacteria on plate count agar (PCA) incubated at 30 and 37°C for 48 h. Culturable bacteria were not found in either the soil extract or in the effluent.

Microcosms (30 ml) of both the soil extract and the lagoon effluent were prepared in triplicate.

### Inoculation of the microcosms

Soil extract and lagoon effluent microcosms were inoculated with *L. monocytogenes* CIP 110868 to reach a final concentration of 5 × 10^8^ CFU/ml. Immediately after inoculation, the microcosms were incubated under static conditions at 21°C for 20 min (T1) and 24 h (T2). The temperature of 21°C was selected to mimic the average summer temperature in piggery lagoons in Brittany. In each treatment, three independent microcosms were inoculated with three independent inocula.

### RNA extraction and purification

Total RNA was extracted from the culture broth (T0) and from the inoculum after 20 min (T1) and 24 h (T2) of incubation. RNAs were immediately stabilized by treating 2 ml of the bacterial cultures with RNA protect bacterial reagent (Qiagen, France) according to the manufacturer's instructions. Three independent replicates were prepared and treated separately. Four RNA extractions were performed of each replicate.

RNA was isolated according to the protocol described by Piveteau et al. (2011). Briefly, bacteria were harvested by centrifugation at 5,500 g for 5 min. Pellets were suspended in 700 ml of RLT buffer supplemented with 1% β-mercaptoethanol and 0.2 g of RNase-free glass beads (100 μm). Cells were mechanically disrupted in a Fast Prep®-24 instrument (MP Bio, France) with four cycles (6 m/s, 30 s). Total RNA was isolated using the Qiagen RNeasy kit (Qiagen, France) according to the manufacturer's instructions. After treatment with DNase I (DNAse on column, 10 U, Qiagen), RNA was eluted in 40 μl of RNase-free water. The four eluates of the same replicate were then pooled and treated with RQ1 RNase free DNase (Promega, France) in RNasin (40 U, Promega). RNAs were purified and concentrated using the RNeasy MinElute cleanup kit (Qiagen, France) according to the manufacturer's instructions. The quality and purity of the RNAs were determined spectrophotometrically and by capillary electrophoresis using the Bioanalyser 2100 (Agilent, France).

All RNAs were stored at −80°C until analysis.

### Sequencing and data analysis

RNA stabilization, 16S, 23S, and 5S RNA depletion, library preparation (“strand specific” method), clustering and sequencing (illumina HiSeq2000) were performed by Sistemas Genómicos (Valence, Spain) according to the manufacturer's instructions. Sequenced reads were quality-checked using FastQC. Reads were mapped against the most recent version of the *L. monocytogenes* EGD-e genome provided by NCBI database, and quantified. The statistical analysis performed by Sistemas Genómicos is described below. The differential expression study between groups of samples was achieved using Python and R statistic packages. The gene differential expression was studied with the algorithm proposed by DESeq2 (Anders and Huber, 2010; Anders et al., 2013) using a negative binomial distribution as dispersion model. Using the functional annotation from Uniprot database (http://www.uniprot.org/), Sistemas Genómicos realized a hyper-geometric test using different statistical packages from statistical platform R to performed the functional enrichment study. T-statistics and *p*-values (FDR adjusted *p*-value of 0.05) were calculated to identify differentially expressed genes. The fold changes at T1 and T2 were calculated by comparing results with expression levels at time 0.

Genes with at least a three-fold change were considered significantly different in the interpretation applied in this study. Finally, functional studies and differential analyses were performed between samples. Genes differentially transcribed were grouped according to their functional category (Glaser et al., 2001).

## Results and discussion

**Figure 1:**
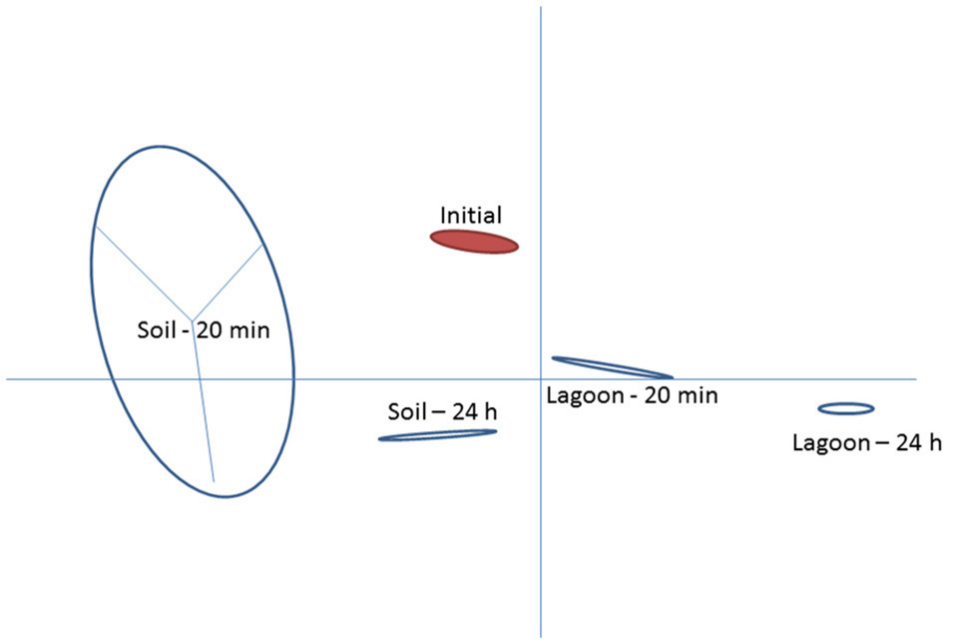
Principal component analysis of *Listeria monocytogenes* transcriptomes after 20 min and 24 h of incubation in lagoon effluent and soil extract.

This study investigated variations in the transcriptome of a strain of *L. monocytogenes* isolated from pig manure after transfer to lagoon effluent or to soil extract to better understand the mechanisms behind its physiological adaptation to these environments. The two matrices selected were (i) a lagoon filled with biologically treated manure and (ii) soil located in the vicinity of the lagoon, which may receive manure containing *L. monocytogenes*. Raw reads ranged between 3.1 × 10^7^ and 3.8 × 10^7^ in all samples. After removing low quality reads, the average number of properly paired mapped reads ranged between 1.9 × 10^7^ and 2.9 × 10^7^ for the datasets from cells transferred in lagoon effluent (62.8–76.7% of properly paired reads), and from 3.0 × 10^7^ to 3.6 × 10^7^ for the datasets from cells transferred in soil extract (92.2–95.8% properly paired reads). Among the differentially expressed entries (with a fold change ≥2), the total number of genes which had mapped reads was 2,267 for the 4 conditions (soil and lagoon at 20 min and 24 h). As shown in Figure 1, each condition tested led to differences in transcriptomes. PCA also indicated homogeneity within each condition except after 20 min in the soil.

**Figure 2:**
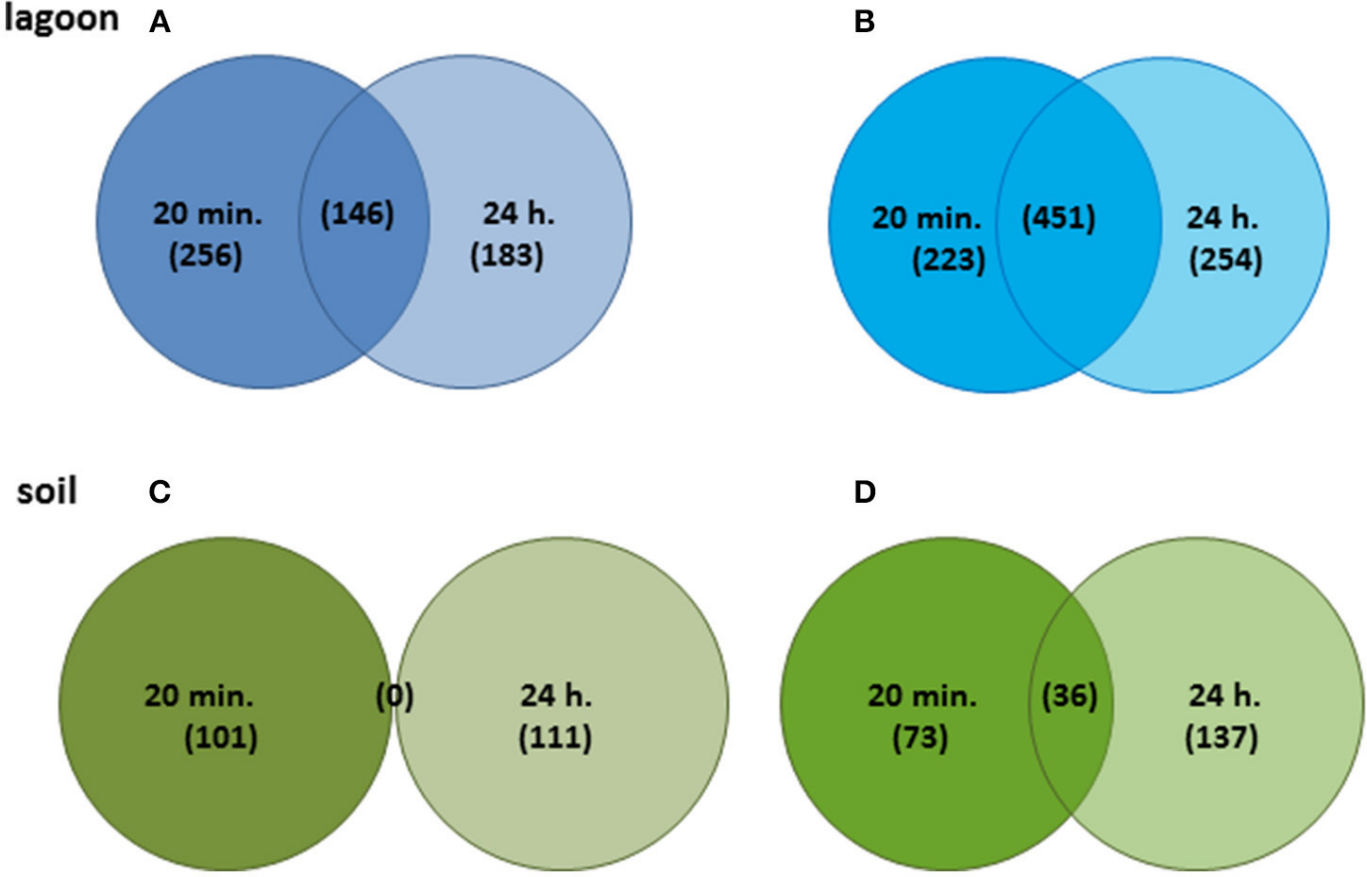
Venn diagrams of genes showing significant differences after 20 min and 24 h. **(A)** Genes with higher transcript levels in the lagoon effluent, **(B)** genes with lower transcript levels in the lagoon effluent, **(C)** genes with higher transcript levels in the soil extract, **(D)** genes with lower transcript levels in the soil extract.

The variations of transcriptome were higher in lagoon than in soil. A total of 1,513 genes were differentially transcribed in the lagoon effluent, 585 with higher and 928 genes with lower transcript levels. In the soil, 458 genes were differentially expressed of which 212 with higher and 246 genes with lower transcript levels (Figure 2). While the percentage of properly paired reads supports a comprehensive overview of the transcriptome variations during incubation in soil, the fraction of unexplained changes was higher in the lagoon effluent as suggested by the lower percentage of paired reads. A total of 1,513 genes were differentially transcribed in the lagoon effluent, 585 with higher and 928 genes with lower transcript levels. In the soil, 458 genes were differentially expressed of which 212 with higher and 246 genes with lower transcript levels (Figure 2). Interestingly, the values of fold changes observed in differentially expressed genes were lower in the soil than in the lagoon effluent (Table 1). Indeed, in the soil, fold changes went from −14.7 to +48.3, whereas in the lagoon effluent, they went from −19,267 to +2,146.

**Table 1:** Number of genes highly differentially expressed in the different conditions.

**Fold change**	**Lagoon up**		**Lagoon down**		**Soil up**		**Soil down**	
	**20 min**	**24 h**	**20 min**	**24 h**	**20 min**	**24 h**	**20 min**	**24 h**
>10	77	114	167	232	3	24	–	11
>100	2	22	130	12	–	–	–	–
>1,000	–	1	47	–	–	–	–	–
>10,000	–	–	2	–	–	–	–	–

## Marked variations in the transcriptome detected in lagoon effluent

### Overview

The transfer of *L. monocytogenes* CIP 110868 into the lagoon effluent caused substantial changes of the transcriptome. Within 20 min, 1,076 genes were differentially transcribed. After 24 h, the number of up or down regulated genes remained stable, with 1,034 genes identified with a different transcript level, indicating that transcriptome reshaping took place within minutes following contact with the effluent (Table S1). A high proportion of operons were differentially transcribed (Table 2). Seventy-four complete operons including 218 genes were differentially expressed after 20 min of incubation, which represents 20% of the set of genes with different transcript levels. After 24 h, the proportion increased to 26% with 76 operons including 268 genes. A large set of transcriptional regulators was differentially transcribed at T1 and T2 (functional category 3.5.2, Table 3, Table S1). Among the 209 regulators described in the genome of *L. monocytogenes* EGD-e (Glaser et al., 2001), 76 (36%) were differentially transcribed after 20 min and 83 (40%) after 24 h. Interestingly, most had a lower transcript level. These results confirm the pivotal role of the extensive regulatory repertoire in the ability of *L. monocytogenes* to survive in various environments and to adapt its physiology (Gandhi and Chikindas, 2007). Therefore, it is not surprising that a high proportion of transcriptional regulators and response regulators was detected in the set of differentially transcribed genes. Table 4 lists the differentially transcribed regulators for which biological functions have been described. Similarly, high proportions of the regulons (from 31.4 to 59.4%) of sigma factors and other transcriptional regulators were differentially transcribed after 20 min and 24 h (Table 5). This suggests these regulons may be important for adaptation to the lagoon environment.

**Table 2:** Number of genes, operons and ncRNA differentially transcribed in the lagoon effluent and in the soil extract.

	**20 min**			**24 h**		
	**+**	**−**	**Total**	**+**	**−**	**Total**
**LAGOON EFFLUENT**						
Genes	402	674	1076	329	705	1034
Operons (genes)	16 (44)	58 (174)	74 (218)	17 (120)	59 (148)	76 (268)
ncRNA	14	28	42	9	29	38
**SOIL EXTRACT**						
Genes	101	109	210	111	173	284
Operons (genes)	13 (32)	4 (10)	17 (42)	7 (42)	7 (16)	14 (58)
ncRNA	6	14	20	0	39	39

**Table 3:** Distribution of the genes differentially transcribed in the lagoon effluent in the different functional categories.

**Functional categories (total number of genes included in the category)**	**20 min**		**24 h**	
	**+^a^**	**−^b^**	**+^c^**	**−^d^**
**1. Cell envelope and cellular processes (620)**				
1.1. Cell wall (72)	11	25	6	22
1.2. Transport/binding proteins and lipoproteins (331), including:	67	58	53	51
Phosphotransferase system	29	13	19	5
ATP-binding cassette transporter	30	24	23	24
1.3. Sensors (16)	5	2	2	2
1.4. Membrane bioenergetics (48)	1	9	0	13
1.5. Mobility and chemotaxis (30)	0	1	28	0
1.6. Protein secretion (15)	4	1	1	3
1.7. Cell division (23)	6	1	6	1
1.8. Cell surface proteins (63)	3	29	6	27
1.9. Soluble internalin (4)	0	4	0	1
1.10. Transformation/competence (18)	6	2	4	3
**2. Intermediary metabolism (615)**				
2.1. Metabolism of carbohydrates and related molecules (252) including:	40	41	63	36
2.1.1. Specific pathways (223)	32	39	57	33
2.1.2. Main glycolytic pathways (25)	5	2	4	2
2.1.3. TCA cycle (4)	3	0	0	1
2.2. Metabolism of amino acids and related molecules (149)	32	23	40	23
2.3. Metabolism of nucleotides and nucleic acids (63)	6	2	4	3
2.4. Metabolism of lipids (53)	15	9	6	9
2.5. Metabolism of coenzymes and prosthetic groups (92)	15	14	17	12
2.6. Metabolism of phosphate (6)	3	1	1	2
**3. Information pathways (468)**				
3.1. DNA replication (22)	8	3	1	5
3.2. DNA restriction/modification and repair (36)	4	11	0	17
3.3. DNA recombination (19)	1	2	0	6
3.4. DNA packaging and segregation (14)	0	2	1	5
3.5. RNA synthesis (227), including:	33	52	10	80
3.5.1. Initiation (5)	0	2	0	2
3.5.2. Regulation (209)	28	49	10	75
3.5.3. Elongation (9)	5	0	0	1
3.5.4. Termination (4)	0	1	0	2
3.6. RNA modification (25)	4	1	2	2
3.7. Proteinsynthesis (97)	7	6	3	8
3.8. Protein modification (21)	2	5	0	3
3.9. Proteinfolding (7)	0	3	1	3
**4. Other functions (149)**				
4.1. Adaptation to atypical conditions (39)	6	9	7	15
4.2. Detoxification (22)	1	9	1	10
4.3. Phage-related functions (49)	0	42	2	34
4.4. Transposon and IS (23)	0	18	0	6
4.5. Miscellaneous (16)	0	6	0	6
**5. Similar to unknown proteins (747)**				
5.1. From Listeria (58)	4	29	3	19
5.2. From other organisms (689)	96	146	37	188
**6. No similarity (261)**	22	108	24	90

a *Number of genes with higher transcript levels after 20 min of incubation*.

b *Number of genes with lower transcript levels after 20 min of incubation*.

c *Number of genes with higher transcript levels after 24 h of incubation*.

d *Number of genes with lower transcript levels after 24 h of incubation*.

**Table 4:** Regulators differentially transcribed for which biological functions have already been described.

**Proteins**	**Biological functions or description**	**Regulation**	**Lagoon**		**Soil**		**References**
			**20 min**	**24 h**	**20 min**	**24 h**	
**TRANSCRIPTIONAL REGULATORS OR REGULONS**							
BvrA	Beta glucosidase repressor	Down	×	×			Brehm et al., 1999
ccpA	Catabolite repressor	Up	×	×			Behari and Youngman, 1998
glnR	Glutamine synthetase repressor	Up	×		×		Wurtzel et al., 2012
gltC	Glutamate synthaseactivator	Up	×		×		Huang et al., 2013
hrcA	Heat-shock gene repressor	Down	×	×			Hu et al., 2007a; van der Veen and Abee, 2010
LexA	SOS system repressor	Down	×	×			Wurtzel et al., 2012
Lmo0020	PTS system activator	Down		×			Piveteau et al., 2011
Lmo0106	Chininolytic activity regulation (post-transcriptional level)	Down		×			Larsen et al., 2010
Lmo0178	Xylose repressor	Up	×				Wurtzel et al., 2012
Lmo0229	Class III stress genes repressor	Down		×			Chatterjee et al., 2006
Lmo0252	Penicillinase repressor	Down	×	×		×	Wurtzel et al., 2012
Lmo0297	PTS system activator	Down		×			Piveteau et al., 2011
Lmo0326	Murein hydrolase activity regulator	Down	×				Popowska and Markiewicz, 2006
Lmo0445	Virulence	Down	×	×	×		Raengpradub et al., 2008
Lmo0606	Multidrug efflux pump regulator	Down	×	×			Wurtzel et al., 2012
Lmo0630	PTS systems activators	Down	×				Piveteau et al., 2011
Lmo0753	Biofilm production, rhamnoseutilization	Down	×	×			Salazar et al., 2013a,b
Lmo1150	Propanediol catabolism, virulence	Up	×	×			Mandin et al., 2007; Mellin et al., 2013
Lmo1262	Phage related	Up	×				Ouyang et al., 2012
Lmo1478	Oxidative stress, acid tolerance, intracellular growth	Up			×		Supa-amornkul et al., 2016
Lmo1618	Acid tolerance	Down	×	×			Phan-Thanh and Mahouin, 1999; Rea et al., 2004
Lmo1618	Acid tolerance	Up			×		Phan-Thanh and Mahouin, 1999; Rea et al., 2004
Lmo1727	Lactose repressor	Up	×				Wurtzel et al., 2012
Lmo2099	PTS systems activators	Up	×				Wurtzel et al., 2012
Lmo2100	Vitamin B6 activator	Up			×		Belitsky, 2014
Lmo2107	Heat shock	Down	×	×			van der Veen et al., 2007
Lmo2138	PTS systems activators	Up	×	×			Wurtzel et al., 2012
Lmo2200	Hydroxyperoxide resistance regulator	Down		×			Chatterjee et al., 2006
Lmo2324	Anti-repressor bacteriophage A118	Down			×		Wurtzel et al., 2012
Lmo2352	Cystine ABC transport activator	Down		×			Garmyn et al., 2012
Lmo2668	PTS systems activators	Up	×	×			Wurtzel et al., 2012
Lmo2690	Muramidase repressor	Up	×				Chatterjee et al., 2006
Lmo2851	Arabinose	Up	×				Wurtzel et al., 2012
mecA	Competence repressor	Down		×			Borezee et al., 2000
perR	Metal ion resistance	Down		×			Rea et al., 2005; Ledala et al., 2010
prfA	Virulence	Down	×	×	×		Johansson et al., 2002; Scortti et al., 2007; Piveteau et al., 2011
pyrR	Pyrimidine operon	Down				×	Wurtzel et al., 2012
ZurR	Ferric uptake	Up			×		Dowd et al., 2012
**RESPONSE REGULATORS**							
CesR	Antibiotic resistance	Up	×		×		Kallipolitis et al., 2003
cheY	Chemotaxis	Up		×		×	Flanary et al., 1999; Williams et al., 2005
degU	Motility, virulence	Up	×				Knudsen et al., 2004; Williams et al., 2005
Lmo0984	Cell autolysis	Up	×				Bennett et al., 2007
Lmo1022	Cell envelope	Up	×				Fritsch et al., 2011
Lmo1172	Cold adaptation	Up	×	×			Chan et al., 2008
Lmo1745	Cell envelope	Down				×	Williams et al., 2005

**Table 5:** Percentage of genes from sigma factors and other transcriptional regulators (regulons) with significant variations in transcription after 20 min (T1) and 24 h (T2) of incubation in lagoon effluent (Lag) and soil extract (Soil).

**Regulon**	**Lag T1**	**Lag T2**	**Soil T1**	**Soil T2**
σB (216)^a^	36.6 (79)^b^	35.2 (76)	13.4 (29)	4.2 (9)
σC (24)	58.3 (14)	41.7 (10)	0.0 (0)	16.7 (4)
σL (51)	41.2 (21)	45.1(23)	2.0 (1)	23.5(12)
σH (169)	39.1 (66)	47.9 (81)	14.2 (24)	12.4 (21)
PrfA (70)	58.6 (41)	41.4 (29)	17.1 (12)	5.7 (4)
CodY (86)	31.4 (27)	52.3 (45)	2.3 (2)	26.7 (23)
CtsR (64)	40.6 (26)	59.4(38)	12.5 (8)	23.4 (15)

a *The total number of genes in the regulon is given in brackets, according to Milohanic et al. (2003), Bennett et al. (2007), Hu et al. (2007a,b), Hain et al. (2008), and Chaturongakul et al. (2011)*.

b *The number of genes with significant variation in their transcript level is given in brackets*.

### Upregulation of transporters of carbon and energy sources

A large repertoire of differentially transcribed genes illustrated the metabolic adaptation of *L. monocytogenes* CIP 110868 to its environment within the first minutes of incubation (Table 3). Transporters required for the use of available resources were highly represented, suggesting that a readjustment of the transcriptome occurred rapidly in order to access resources available in the lagoon environment. After 20 min of incubation in the lagoon, a total of 125 genes associated with transport were differentially transcribed. This represents 38% of the genes identified in the genome of *L. monocytogenes* EGD-e as being devoted to carbohydrate uptake (Glaser et al., 2001; Fichant et al., 2006). These genes included 42 phosphoenolpyruvate-dependent phosphotransferase systems (PTS systems) and 54 ATP-binding cassette transporters (ABC transporters). Among them, respectively 29 and 30 genes had higher transcript levels. These genes encoded proteins involved in glycerol (*lmo1539*), galactitol (*lmo2667*), fructose (*lmo0039, lmo0400* and *lmo0358*), and maltose-maltodextrin (*lmo2123-24-25*) uptake. Moreover, arabinose and lactose transcriptional regulators (*lmo2851* and *lmo1727*, respectively) and a xylose repressor (*lmo0178*) were identified in the set of genes with higher transcript levels. In addition, three known activators of PTS systems (*lmo2138, lmo2099*, and *lmo2668*) were also found to be upregulated. Genes encoding transporters found in the set of genes with lower transcript levels were mainly related to the transport of metal cations but none to the transport of carbohydrates.

The same pattern was observed after 24 h of incubation, except that the percentage of genes encoding transporters decreased from 38 to 31%.

### Modification of the cell envelope

Throughout the experiment, genes involved in cell surface rearrangement were differentially transcribed. The functional categories “cell wall” and “cell surface proteins” were highly represented. Most of the genes were downregulated. For example, seven genes encoding teichoic acid synthesis had lower transcript levels. Similarly, the transcript level of genes encoding cell surface proteins also decreased. This included peptidoglycan bound proteins and internalins. As reviewed by Bierne and Cossart (2007), the adjustment of the cell envelope is of major importance for coping with new environmental conditions. Cell surface proteins fulfill many functions as diverse as bacterial growth, peptidoglycan metabolism, sensing of and protection from environmental stresses, adhesion and invasion of host cells and signaling. For example, teichoic acids, which are directly linked to the structure of the outer cell wall, enable reinforcement of the cell wall of *L. monocytogenes* when environmental conditions are unfavorable. The products of both genes of the *tagGH* operon are involved in translocation in teichoic acids. The operon, controlled by a σA-dependent promoter, was first described in *Bacillus subtilis* for which it is essential for cell growth (Lazarevic and Karamata, 1995). In *L. monocytogenes*, the operon plays a role in protecting cells against the biocide benzethonium chloride (Casey et al., 2014). Additional genes involved in the biosynthesis and the glycosylation of teichoic acids, such as *ggaB, lmo1077, lmo1085, gtcA, tagB*, and *tagO*, were also found in the set of genes with lower transcript levels. It is important to note that these genes were differentially transcribed with a fold change in the region of −1,000.

The variations in transcript levels observed in these functional categories point to the need for a readjustment of the cell surface to adjust to the new environment.

### Protection of *L. monocytogenes* against antibiotics and metal ions

*lmo0441, pbpA, pbpB, lmo2229*, and *lmo1438* genes were positively transcribed. These penicillin-binding protein encoding genes have been shown to be involved in cell wall functioning (Rismondo et al., 2015), cell division during growth (Krawczyk-Balska et al., 2012) and resistance to β-lactam antibiotics (Van de Velde et al., 2009; Collins et al., 2012). Moreover, the penicillinase repressor *lmo0252* had a lower transcript level, suggesting deactivation of repression and the production of penicillinase. Similarly, the transcriptional repressor (*lmo2690*) from the TetR family, which targets positive regulators of multi-drug efflux pumps, had a lower transcript level, indicating that the repression of the pumps may be lifted in the effluent. We also found the tetracycline resistance protein coding gene *lmo0839* in the set of genes with higher transcript levels. The response regulator *lmo1022*, which resembles the LiaSR two-component system of *B. subtilis* known to be induced when antibiotics interfere with the cytoplasmic membrane (Fritsch et al., 2011), was found to be activated. Finally, *lmo1967* was also activated. *lmo1967*, first described for toxic ion resistance, is required for optimum resistance to nisin and other antimicrobial molecules (oxacillin, cefuroxime, cefotaxime, methicillin, and bacitracin; Collins et al., 2010). These data suggest that mechanisms of protection against antibiotics had been triggered. Conversely, other genes (*lmo0872, lmo1409*, and *lmo1617*), also implicated in antibiotic resistance and in the functioning of multi-drug efflux transporter, were downregulated.

Antibiotics (mainly oxytetracycline and chlortetracycline) are frequently administered to pigs and animals may excrete from 25 to 90% of the administered antibiotics as parent compounds. As a consequence, high concentrations of antibiotics are detected in manures (Qian et al., 2016). For example, oxytetracycline and chlortetracycline have been detected at concentrations in the order of mg per kg of dry weight in manure samples (Widyasari-Mehta et al., 2016). As a result, it is possible that lagoon effluents (liquid fraction of biologically treated manure) are also contaminated by antibiotics and that *L. monocytogenes* had to face these molecules in the lagoon effluent, as our results suggest.

Metal ions are commonly detected in piggery effluents (Xiong et al., 2010; Hölzel et al., 2012). For example in the lagoon effluent used in this study, copper and zinc were detected at concentrations of 16 and 55 mg/L respectively (data not shown). The upregulation of *lmo1478*, a transcriptional regulator of the MerR family, within the first minutes of incubation in the effluent, suggests that *L. monocytogenes* responded to exposure to metal ions. In contrast, genes encoding efflux systems for cadmium/arsenic and for zinc/arsenic transport were downregulated.

### Activation of other stress responses

The introduction of *L. monocytogenes* in harsh environments triggers the regulation of genes involved in stress responses (Piveteau et al., 2011; Casey et al., 2014).

A battery of genes involved in stress response was detected among genes with higher transcript levels throughout the course of the experiment. Briefly, the expression of the cold shock genes *cspD* and *cspL* increased. Upregulation of the *opuC* operon (*opuCA, opuCB, opuCD*) suggests a response to osmotic stress. This system is involved in the uptake of the osmoprotectants glycine-betaine, carnitine, and choline. These molecules have previously been shown to play a role in the osmoregulation and cold adaptation of *L. monocytogenes* (Fraser et al., 2000). The GltC activator also had a higher transcript level, suggesting that oxidative stress occurred in the lagoon environment. The LexA SOS system repressor and the repressor of class III stress genes Lmo0229 were in the set of genes with lower transcript levels. Finally, it is important to note that σB, which plays a central role in the stress response of *L. monocytogenes* and the RsbU phosphatase involved in the regulation of σB, had higher transcript levels. Among the 216 genes of the σB regulon (Hain et al., 2008), 79 were differentially transcribed after 20 min of incubation and 76 after 24 h.

All these modifications suggest that incubation in the lagoon triggered responses to environmental stresses. Conversely, regulators involved in adaptation to heat shock (Lmo2107; van der Veen et al., 2007) and acid shock (Lmo1618; Phan-Thanh and Mahouin, 1999; Rea et al., 2004) were in the set of genes with lower transcript levels. Some of these variations may be explained by the conditions under which inocula were prepared. Indeed, the pH of the broth and temperature used as reference differed from the conditions of the lagoon.

### Activation of amino acid metabolic pathways

The number of genes dedicated to the metabolism of amino acids and related molecules increased with time, from 55 genes differentially transcribed after 20 min to 63 genes after 24 h. These genes are particularly involved in the synthesis of leucine and isoleucine (*ilvDBC, leuABCD, ilvA*), histidine (*hisEIA, hisJ, hisC*), threonine (*thrC*), glutamate and glutamine (*serC, glnA*), alanine (*daI*), glycine (*lmo1349-50, glyA*), arginine (*argFDBJC, argGH*), lysine (*dapF*), and tryptophan (*trpABCD*; Figure S1). In addition, several riboswitches (Tbox, lysine, glycine, TPP, purine, pyrimidine, glmS riboswitch), which regulate amino acid biosynthesis, were also found in the set of upregulated genes (Table S2).

### Motility triggered after 24 h incubation

After 24 h of incubation in the lagoon environment, the genes involved in motility and chemotaxis were highly activated. In this functional category, 28 genes had higher transcript levels after 24 h but not after 20 min. Among the 28 genes, we identified the well-described *flaA* encoding flagellin (Grundling et al., 2004), *motAB* encoding flagellar motor rotation proteins (Michel et al., 1998) and *fli, flg*, and *flh* genes. Interestingly, these genes belong to two operons, all of whose genes were also activated (42 genes). Among them *cheYA* encodes the two component system responsible for the regulation of chemotaxis in *L. monocytogenes* (Flanary et al., 1999; Williams et al., 2005). Motility may be a strategy used by cells to cope with their environment. Several of these genes (for example *flaA* and *motAB*) are thermoregulated, with higher expression at 25°C (saprophyte life in the environment) and lower expression at 37°C (intracellular life; Michel et al., 1998; Grundling et al., 2004).

### Virulence genes downregulated

As for some genes involved in motility, temperature is an environmental cue that affects PrfA activity and expression of its regulon (Freitag et al., 2009; de las Heras et al., 2011). During the saprophytic lifestyle of the bacteria (at temperatures below 30°C), the secondary structure of the mRNA of *prfA* forms a hairpin structure which masks the ribosome binding region. In this conformation, the thermosensor represses the translation of *prfA* mRNAs. *prfA* was downregulated throughout the course of our experiment. Among the 70 genes referenced in the PrfA regulon (Milohanic et al., 2003; Chaturongakul et al., 2011), 41 had different transcript levels after 20 min and 29 after 24 h. As a result of introducing *L. monocytogenes* in an environmental matrix, genes specifically involved in the adaptation of the pathogen to the intracellular environment were downregulated. Internalin-coding genes *inlB, inlC, inlE, inlG*, and *inlH* were in the set of genes with lower transcript levels. Similarly, *lmo0754, lmo2067*, and *lmo0446*, which encode bile acid hydrolases in response to acid stress, *lmo0445*, a candidate regulator of virulence genes (Raengpradub et al., 2008) and *lmo2200*, an ortholog regulator of *ohrR* in *B. subtilis*, which is essential for intracellular growth (Chatterjee et al., 2006) were downregulated.

### Genes in the “transformation/competence” category upregulated

Genes of the ComG (*comGA* and *comGB*) and ComE (*comEB* and *comEC*) operons were found to have a higher transcript level in the lagoon environment both at 20 min and 24 h. They are similar to *B. subtilis comG* and *comE* operons, which encode proteins enabling he development of competence and the acquisition of extracellular DNA (Dubnau, 1997). The MecA regulatory protein, which represses competence genes (Borezee et al., 2000), was in the set of genes with lower transcript levels. While the Com master activator is disrupted by a *Listeria*-specific prophage in some strains including EGD-e, other strains harbor the full version of *comK*. Although competence has never been demonstrated in *L. monocytogenes*, Rabinovich et al. (2012) showed that excision of the prophage can allow functional transcription of *comK* and that the role of Com systems in *Listeria* might have diverged and could be useful for phagosome escape and virulence. Alternatively, competence may be possible under some environmental conditions yet to be identified such as lagoons. Some isolates might be competent even though signals inducing competence have not been identified (Buchrieser et al., 2003).

### Downregulation of ncRNAs after incubation in the lagoon effluent

Whatever the incubation time, a wide range of ncRNAs was detected in the sets of differentially transcribed targets (Table S2) most of which presented a lower transcript level. Like in other bacterial models, ncRNAs play a central role in the adaptation of *L. monocytogenes* to its environment. So far, a role for the non-coding part of the genome has been reported in the regulation of virulence (Mandin et al., 2007; Loh et al., 2009; Toledo-Arana et al., 2009; Mraheil et al., 2011; Nielsen et al., 2011; Mellin and Cossart, 2012), in the transport and metabolism of sugars (Mandin et al., 2007; Nielsen et al., 2011), in the transport of metal ions (Wurtzel et al., 2012), in the synthesis of vitamins (Mellin and Cossart, 2012), in the formation of biofilm (Peng et al., 2016) and in the regulation of motility (Mellin and Cossart, 2012). In our study, we found that Rli28, Rli31, Rli38, Rli49, and Rli50, which are involved in invasion of the host's blood system, in the regulation of virulence factors (Toledo-Arana et al., 2009), in intracellular multiplication in mouse macrophage P338D1 and complete virulence in *Galleria mellonella* models (Mraheil et al., 2011), had lower transcript levels after incubation in the lagoon environment. Similarly, the virulence regulators LhrC1, LhrC2, LhrC3, and LhrC4 riboswitches were deactivated. Conversely, Rli60 was found in the set of ncRNAs with higher transcript levels. This ncRNA has recently been reported to play a role in the response (growth and biofilm formation) of *L. monocytogenes* EGD-e to environmental stresses (temperature, pH, osmotic pressure, alcohol; Peng et al., 2016).

### Highly differentially transcribed genes

To extract more information on the response of *L. monocytogenes* to the lagoon environment, we next focused on genes for which at least 10-fold increase was observed. A total of 568 genes met this criterion (fold change >10 = 183 genes and fold change < −10 = 385 genes). Among the genes with a 10-fold higher transcript level, functional categories 1.2 (transport/binding proteins and lipoproteins), 2.1.1 (specific pathways), 3.5.2 (regulation), and 5.2 (similar to unknown proteins from other organisms) were the most widely represented. Interestingly, the genes *lmo0049* (*agrD*) and *lmo0050* (*agrC*), encoding respectively the propeptide AgrD (precursor of the auto-inducing peptide) and the histidine kinase AgrC, were in this set of genes. AgrD and AgrC are a part of the Agr communication system (Rieu et al., 2007). This indicates that communication between bacteria might have been induced in the lagoon environment. As the Agr communication system is involved in the overall adaptation of the cell (Rieu et al., 2007; Riedel et al., 2009; Vivant et al., 2014) by integrating environmental cues, it was not surprising to find a marked increase in the transcript levels of the *agrD* and *agrC* genes. The functional categories highly represented in the set of genes with fold changes lower than −10 differed. We identified categories 3.5.2 (regulation), 4.3 (phage-related functions), 5.2 (similar to unknown proteins in other organisms), and 6 (no similarity). Genes with phage-related functions are often described in studies involving differential analysis (Piveteau et al., 2011; Casey et al., 2014). As described in the study by Piveteau et al. (2011), transcript levels of phage-related genes decreased within the first minutes of incubation (42 of the 48 phage-related genes at 20 min) and throughout the course of the experiment (36 genes at 24 h). However, the function of these modifications is still not known. In our study, the majority of the genes in the “phage-related functions” category were related to the Listeria phage A118.

### The transfer of *L. monocytogenes* in soil led to a slight modification of its transcriptome

Smaller variations in the transcriptome were observed after incubation of *L. monocytogenes* CIP 110868 in the soil than in the lagoon (Table S3). We detected 210 genes with different transcripts level after 20 min of incubation and 284 genes after 24 h of incubation in the soil extract (Table 2, Figures 2C,D). Another difference between the sets of differentially transcribed genes from lagoon and soil is that, after incubation of *L. monocytogenes* CIP 110868 in soil extract, fold changes were low (Table 1). Indeed, of the 210 differentially transcribed genes at 20 min, only three had fold changes >10 (one transporter and two unknown proteins). On average, fold changes ranged from −3.5 to 4.4. A similar pattern was observed after 24 h of incubation, when 35 of the 284 genes were highly differentially transcribed (fold change >10 or < −10). These genes were mainly dedicated to transport, mobility and unknown functions.

As described in the lagoon environment, complete operons were differentially transcribed during incubation in soil extract (Table 2). Transcription and response regulators were differentially transcribed but their proportion was smaller than in the lagoon environment. The percentage of regulators differentially transcribed was 5.7 and 4.6% of the genes differentially transcribed at 20 min and 24 h in the soil, whereas these proportions were 7.1 and 8% in the lagoon. The proportion of genes under the control of regulators and sigma factors was also smaller. Interestingly, the proportion of genes of the σC, σL, and CodY regulons in the set of differentially transcribed genes increased after 24 h (Table 5).

Surprisingly, genes with higher transcript levels differed completely at T1 and T2 (Figure 2C). In contrast, 37 genes with lower transcript levels were common at the two incubation times. They encoded unknown proteins and the internalin InlC.

### Motility

Like in the lagoon environment, activation of a large panel of genes involved in motility was observed after 24 h of incubation (Table 6), including the regulator CheY. This confirms that motility is a strategy used by *L. monocytogenes* during its saprophytic lifestyle. For example, motility offers the opportunity to access a wide range of nutrients.

**Table 6:** Distribution of the genes differentially transcribed in the soil extract in the functional categories.

**Functional categories (total number of genes included in the category)**	**20 min**		**24 h**	
	**+^a^**	**−^b^**	**+^c^**	**−^d^**
**1. Cell envelope and cellular processes (620)**				
1.1. Cell wall (72)	2	0	0	0
1.2. Transport/binding proteins and lipoproteins (331), including:	14	3	27	2
Phosphotransferase system	1	0	11	2
ATP-binding cassette transporter	8	1	13	0
1.3. Sensors (16)	1	0	1	0
1.4. Membrane bioenergetics (48)	0	1	1	1
1.5. Mobility and chemotaxis (30)	0	0	29	0
1.6. Protein secretion (15)	3	0	0	2
1.7. Cell division (23)	3	0	1	1
1.8. Cell surface proteins (63)	0	1	2	1
1.9. Soluble internalin (4)	0	1	0	1
1.10. Transformation/competence (18)	0	0	0	0
**2. Intermediary metabolism (615)**				
2.1. Metabolism of carbohydrates and related molecules (252), including:	2	5	18	1
2.1.1. Specific pathways (223)	1	4	18	1
2.1.2. Main glycolytic pathways (25)	1	1	0	0
2.2. Metabolism of amino acids and related molecules (149)	12	3	1	3
2.3. Metabolism of nucleotides and nucleic acids (63)	7	0	0	1
2.4. Metabolism of lipids (53)	3	0	1	0
2.5. Metabolism of coenzymes and prosthetic groups (92)	1	0	3	0
2.6. Metabolism of phosphate (6)	0	1	0	1
**3. Information pathways (468)**				
3.1. DNA replication (22)	0	0	0	1
3.2. DNA restriction/modification and repair (36)	3	2	0	2
3.4. DNA packaging and segregation (14)	0	0	0	1
3.5. RNA synthesis (227), including:	9	4	2	12
3.5.2. Regulation (209)	8	4	2	11
3.5.4. Termination (4)	1	0	0	1
3.6. RNA modification (25)	2	0	0	0
3.7. Protein synthesis (97)	3	1	0	6
3.8. Protein modification (21)	0	0	0	1
3.9. Protein folding (7)	0	1	0	0
**4. Other functions (149)**				
4.1. Adaptation to atypical conditions (39)	1	3	0	4
4.2. Detoxification (22)	0	2	1	2
4.3. Phage-related functions (49)	0	32	4	1
4.4. Transposon and IS (23)	0	1	0	2
4.5. Miscellaneous (16)	0	3	2	1
**5. Similar to unknown proteins (747)**				
5.1. From Listeria (58)	0	3	0	13
5.2. From other organisms (689)	29	21	9	50
**6. No similarity (261)**	6	21	9	63

a *Number of genes with higher transcript levels after 20 min of incubation*,

b *Number of genes with lower transcript levels after 20 min of incubation*,

c *Number of genes with higher transcript levels after 24 h of incubation*,

d *Number of genes with lower transcript levels after 24 h of incubation*.

### Transport/binding proteins

Incubation of *L. monocytogenes* for 20 min in the soil extract led to the deactivation of transporters dedicated to fructose, sorbitol, and ribulose and the activation of ABC transporters of metal ions. Moreover, transporters of spermidine and putrescine were also present in the set of genes with higher transcript levels. These two polyamines are involved in numerous biological processes and are essential for biofilm formation (Burrell et al., 2010; Wortham et al., 2010; Sakamoto et al., 2012). As confirmation that *L. monocytogenes* was introduced in a natural environment, a PTS system dedicated to the transport of lichenan, a polysaccharide isolated from lichen, was also activated.

The proportion of genes from category transport and binding protein was higher at 24 h (Table 6). Our results illustrate the requirement to acquire substrates available in the soil extract. This included plant residues such as cellobiose (*lmo2708, lmo2762-63* and *lmo2765*), mannitol (*lmo2651*), maltose and maltodextrin (*lmo2122-26*), rhamnose, and rhamnulose (*lmo2847-49*), residues of arthropods and fungi such as chitin (*lmo0105* and *lmo1883*) and residues of algae and plant exudates such as galacticol (*lmo2665-67*). The upregulation of transporters to acquire substrates from various sources in soil has already been described (Piveteau et al., 2011).

### Stresses encountered

Lesser variations of transcripts of genes related to stress response were detected from soil than from the lagoon environment, as only a few genes dedicated to stress response were differentially transcribed. They are mainly involved in the adaptation of *L. monocytogenes* to changes in temperature. Broth and incubation temperatures differed by about 10°C in our experiment, which explains the response to thermal stimuli. We identified as downregulated the genes encoding the cold shock proteins CspB and CspL and the heat shock protein GroES. The transcriptional regulator NmlR involved in oxidative stress and in tolerance to acid (Supa-amornkul et al., 2016) was found to be activated while the general stress protein encoded by *lmo1601* was downregulated. *L. monocytogenes* is ubiquitous in the environment. The pathogen has been detected in soils (Locatelli et al., 2013a) and its ability to survive in this environment for long periods has been repeatedly demonstrated (Dowe et al., 1997; Moshtaghi et al., 2009; McLaughlin et al., 2011; Locatelli et al., 2013b).

### Virulence and intracellular determinants

The virulence master regulator PrfA, the internalin InlC, and a bile acid hydrolase (*lmo2067*) were downregulated 20 min after *L. monocytogenes* was introduced in the soil extract. This is consistent with the saprophytic lifestyle of the bacteria. These observations are consistent with the observations made by Piveteau et al. (2011). In their study, transcript levels of virulence determinants including PrfA and internalins decreased 18 h after *L. monocytogenes* was transferred in soil extract microcosms.

### ncRNAs

As confirmation that virulence factors were downregulated within the first minutes of incubation, ncRNAs involved in the regulation of virulence (Rli31, Rli50, and Rli47) were in the set of ncRNAs with lower transcript levels after 20 min of incubation (Table S4). Rli31 and Rli50 are required for full virulence in murine, larva, and macrophage infection models (Mraheil et al., 2011), and Rli47 (*sreB*) is a trans-acting ncRNA which controls the expression of the regulator PrfA by binding the untranslated region of its mRNA. Seven ncRNAs were found at both incubation times (Table S4) and all had lower transcript levels. Their functions are not yet described in the literature, except for RliI, which is involved in the regulation of the metabolism and the transport of carbohydrates (Mandin et al., 2007). Interestingly, after 24 h, no ncRNAs with higher transcript levels were detected, while a larger proportion had lower transcript levels. Among them, we mainly identified the four LhrC (1, 2, 3, and 4) riboswitches and Tbox and SAM riboswitches.

### Phage-related functions

As observed in the lagoon effluent, several genes from functional category phage-related functions were in the set of genes with lower transcript levels within the first minutes of incubation in the soil extract (Table 6). These targets matched with genes coding proteins from bacteriophage A118.

### Similar readjustments of the transcriptome of *L. monocytogenes* in the lagoon effluent and in the soil

**Figure 3:**
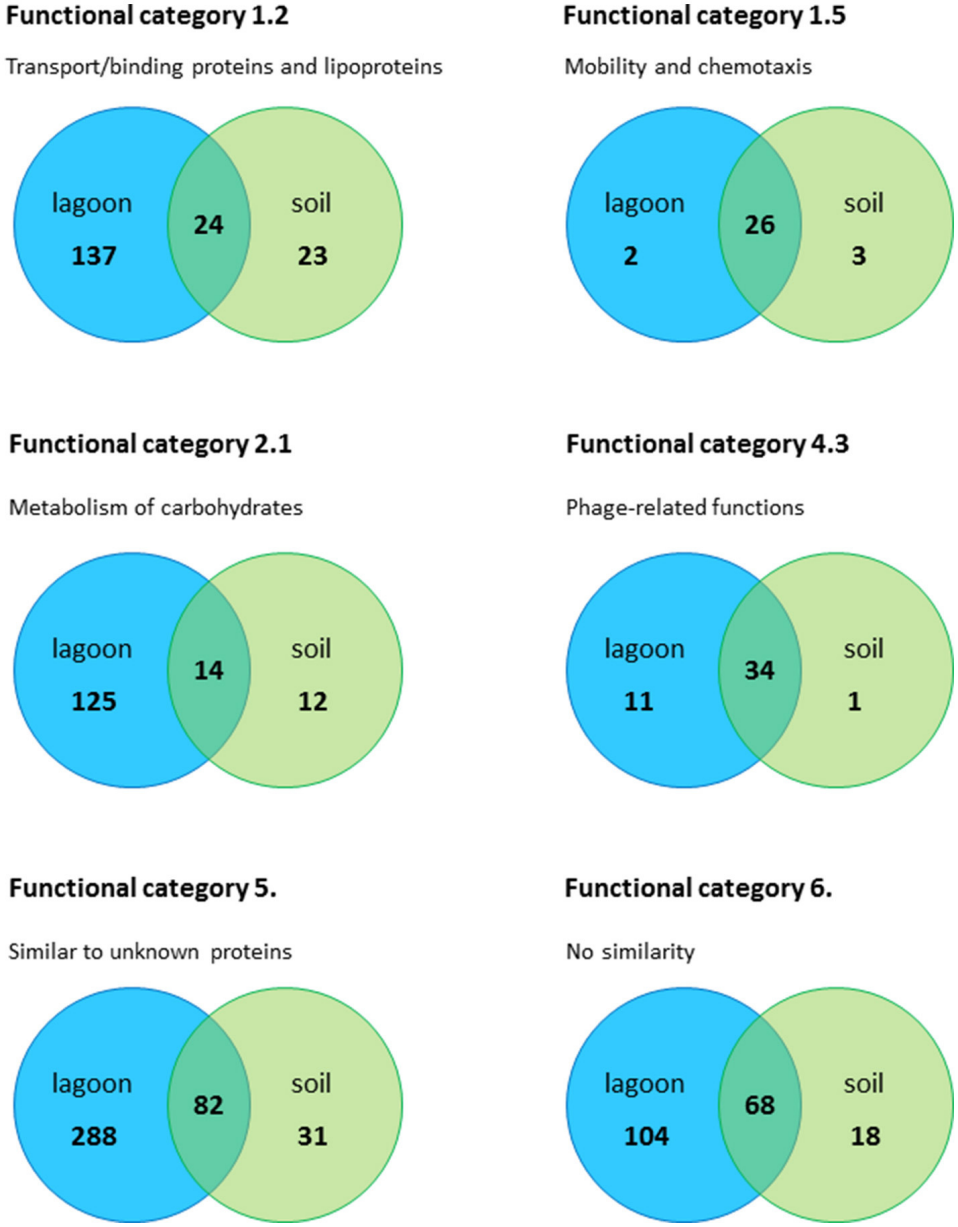
Number of genes in the lagoon effluent and in the soil extract, distributed in the most important functional categories.

Regardless of the incubation time (20 min and 24 h), a large fraction of the genes differentially expressed in the soil extract (42–78%) were shared with the lagoon effluent (Figure S2). The adaptation of *L. monocytogenes* CIP 110868 to the natural environment led to similar readjustments of the transcriptome (Figure 3). Whether *L. monocytogenes* was inoculated in the lagoon effluent or in the soil extract, genes encoding proteins involved in motility and chemotaxis and in the transport of carbohydrates were the most widely represented in the set of genes with higher transcript levels. This is commonly reported in the literature, except that nutrients acquired by bacteria differ depending on the environment. Within genes with lower transcript levels, in both conditions, we mainly found genes in the “phage-related functions” category. It is worth mentioning that a large proportion of the genes differentially transcribed after the introduction of *L. monocytogenes* CIP 110868 in the natural matrices, encoded proteins with unknown functions. This represents from 25% of the set of genes with higher transcript levels to 60% of the set of genes with lower transcript levels. Finally, the proportion of genes from the PrfA, CodY, and CtsR regulons increased between 20 min and 24 h both in the lagoon and in the soil.

### Genes from the category cell envelope were specifically represented in the lagoon effluent

Comparison of differentially transcribed genes in soil and lagoon led to the identification of specific variations. Three functional categories (cell wall, competence, and metabolism of amino acids) were highly represented in the lagoon environment but not in the soil. Differences in the composition of the two matrices could explain such results. For example, the higher concentrations of detrimental molecules such as antibiotics and metal ions in lagoon effluents might trigger upregulation of genes leading to the adjustment of the cell wall for protection against toxic compounds.

## Conclusion

In this study, we showed that the response of *L. monocytogenes* CIP 110868 differed according to the matrix and changed over time. Although, expression profiles fluctuated in the lagoon effluent and in the soil extract, similar mechanisms of adaptation were involved. The adaptation of strain CIP 110868 resulted in the activation of (i) genes encoding transport systems, (ii) genes encoding enzymes dedicated to the assimilation of carbohydrates and the production of energy and (iii) genes involved in motility and chemotaxis of the cells. Finally, we observed a higher proportion of differentially transcribed genes in the lagoon effluent than in the soil, suggesting that lagoon-specific environmental cues trigger multiple regulation routes required to coordinate the appropriate response to this environment.

## Author contributions

AP, PP, and JD designed laboratory work. JD performed all laboratory work. AP, PP, JD, and AV analyzed and interpreted of transcriptomic data. All authors co-wrote the manuscript. AV drafted the manuscript and AP finalized the manuscript.

### Conflict of interest statement

The authors declare that the research was conducted in the absence of any commercial or financial relationships that could be construed as a potential conflict of interest.

## Supplementary material

Table S1 Genes differentially expressed in the lagoon effluent.

Table S3 Genes differentially expressed in the soil extract.
