# Supplementary material for: Transcriptomic Analysis of the Adaptation of Listeria monocytogenes to Lagoon and Soil Matrices Associated with a Piggery Environment: Comparison of Expression Profiles
Source: Front Microbiol. 2017 Sep 26;8:1811. doi: 10.3389/fmicb.2017.01811 (PMC5623016; doi:10.3389/fmicb.2017.01811)
Supplement: Supplementary file 3 [file Table2.PDF]

Table S2. Location, flanking genes, and fold change in expression of the ncRNAs found to be differentially transcribed in the lagoon effluent.

	Position		Flanking gene		Fold
**ncRNA**	**Start**	**Stop**	**5'**	**3'**	**change**
**ncRNA with higher transcript levels at 20 minutes**					
Rli24	271029	271186	*lmo0256*	*lmo0257*	3.1
Tbox riboswitch	1676392	1676617	*trpE*	*lmo1634*	3.1
SAM riboswitch	1739491	1739597	*lmo1681*	*lmo1682*	3.2
Tbox riboswitch	1642603	1642811	*tyrS*	*ccpA*	3.4
Rli51	207589	207709	*hly*	*mpl*	3.7
Lysine riboswitch	826486	826683	*lmo0798*	*lmo0799*	4.1
Tbox riboswitch	1494104	1494276	*glyQ*	*recO*	4.5
Tbox riboswitch	1597218	1597448	*thrS*	*dnaI*	6.2
glmS riboswitch	756458	756652	*lmo0726*		6.8
Tbox riboswitch	258548	258760	*gltX*	*cysE*	7.7
Tbox riboswitch	1676666	1676907	*trpE*	*lmo1634*	8.3
Glycine riboswitch	1372840	1372931	*comGA*	*gcvT*	8.8
SAM riboswitch	2491058	2491176	*lmo2419*	*lmo2420*	9.6
Tbox riboswitch	1553972	1554200	*hisS*	*lmo1521*	11.1
**ncRNA with lower transcript levels at 20 minutes**					
Rli46	2154765	2155058	*lmo2074*	*lmo2075*	-3.8
sRNA LhrA	2346166	2346434	*lmo2257*		-3.8
purine riboswitch	611017	611119	*lmo0573*	*lmo0574*	-4.6
SAM riboswitch	137133	137235	*lmo0134*	*lmo0135*	-6.8
RliA	513584	513807	*lmo0476*	*lmo0477*	-14.9
Rli23	172171	172268	*lmo0172*		-17.2
**ncRNA with higher transcript levels at 24 hours**					
yybP-ykoY riboswitch	1021463	1021588	*lmo0990*	*lmo0991*	3.4
Rli56	1199859	1199859	*pduQ*	*lmo1172*	3.7
Tbox riboswitch	1809817	1810057	*lmo1740*	*lmo1741*	4.0
Rli52	552327	552421	*lmo0517*	*lmo0518*	4.5
RliA	513584	513807	*lmo0476*	*lmo0477*	4.9
sRNA ssrA/tmRNA	2509854	2510220	*lmo2443*	*lmo2444*	12.9
6S/SsrS RNA	1546343	1546531	*lmo1513*	*lmo1514*	13.0
sRNA rnpB/RNase P	1961804	1962188	*lmo1887*	*lmo1888*	53.9
Rli47	2226024	2226532	*lmo2141*	*lmo2142*	250.5
**ncRNA with lower transcript levels at 24 hours**					
Tbox riboswitch	2823255	2823477	*serS*	*lmo2748*	-3.8
Rli32	600604	600750	*lmo0560*	*lmo0561*	-4.1
Rli41	1276713	1277207	*lmo1252*	*lmo1253*	-5.0
TPP riboswitch	1461163	1461273	*lmo1429*	*lmo1430*	-5.4
pyrR riboswitch	1918154	1918262	*pyrR*	*lmo1841*	-5.6
sRNA LhrC2	232086	232197	*cysK*	*sul*	-9.3
sRNA LhrC1	231884	231994	*cysK*	*sul*	-14.5
**ncRNA with higher transcript level at 20 minutes and 24 hours**					
Tbox riboswitch	2668833	2669082	*lmo2586*	*lmo2587*	7.4
TPP riboswitch	340086	340191	*lmo0314*	*lmo0315*	11.8
Rli39	1179807	1179993	*lmo1149*	*lmo1150*	12.4
SAM riboswitch	882770	882867	*lmo0844*	*lmo0845*	17.1
Rli60	2054124	2054308	*lmo1982*	*ilvD*	18.0
RliH	1180826	1181254	*lmo1150*	*lmo1151*	21.4
**ncRNA with lower transcript level at 20 minutes and 24 hours**					
Rli26	388520	388707	*lmo0360*	*lmo0361*	-3.7
Rli38	1152549	1152917	*lmo1115*	*lmo1116*	-4.9
Rli54	1078584	1079111	*lmo1051*	*pdhA*	-5.5
Rli31	597812	597926	*lmo0558*	*lmo0559*	-6.0
RliC	1154309	1154671	*lmo1117*	*lmo1118*	-6.0
FMN riboswitch	2020487	2020609	*lmo1945*	*lmo1946*	-7.0
RliB	544357	544716	*lmo0509*	*lmo0510*	-7.7

Table S2 (continued). Location, flanking genes, and fold change in expression of the ncRNAs found to be differentially transcribed in the lagoon effluent.

**ncRNA with lower transcript level at 20 minutes and 24 hours**					
Rli49	2660179	2660364	*lmo2579*	*lmo2580*	-7.8
Rli61	2275258	2275363	*lmo2187*	*lmo2188*	-7.9
Rli37	907576	907832	*lmo0866*	*lmo0867*	-14.0
prfA riboswitch	204339	204468	*prfA*	*plcA*	-18.9
Rli53	955829	956001	*lmo0918*	*lmo0919*	-19.6
Rli48	2361274	2361423	*lmo2271*	*lmo2272*	-21.6
sRNA LhrC4	232492	232605	*cysK*	*sul*	-40.9
Rli33	708326	708860	*lmo0671*	*lmo0672*	-42.0
sRNA LhrC3	232289	232400	*cysK*	*sul*	-48.2
Rli50	2783098	2783274	*lmo2709*	*lmo2710*	-58.7
RliG	2386715	2386992	*lmo2302*	*lmo2303*	-64.3
Rli29	507450	507643	*lmo0470*	*lmo0471*	-72.6
Rli44	2039087	2039375	*lmo1964*	*lmo1965*	-74.9
Rli28	507206	507394	*lmo0470*	*lmo0471*	-197.6
Rli62	2364337	2364508	*lmo2277*	*lmo2278*	-301.7
