# Supplementary material for: Transcriptomic Analysis of the Adaptation of Listeria monocytogenes to Lagoon and Soil Matrices Associated with a Piggery Environment: Comparison of Expression Profiles
Source: Front Microbiol. 2017 Sep 26;8:1811. doi: 10.3389/fmicb.2017.01811 (PMC5623016; doi:10.3389/fmicb.2017.01811)
Supplement: Supplementary file 4 [file Table4.PDF]

Table S4. Location, flanking genes, and fold change in expression of the ncRNAs found to be differentially transcribed in the soil extract.

	**Position**		**Flanking gene**		**Fold**
**ncRNA**	**Start**	**Stop**	**5'**	**3'**	**change**
**ncRNA with higher transcript levels at 20 minutes**					
SAM riboswitch	137133	137235	*lmo0134*	*lmo0135*	3.0
PyrR riboswitch	1917471	1917574	*pyrP*	*pyrR*	3.0
SAM riboswitch	2491058	2491176	*lmo2419*	*lmo2420*	3.6
Tbox riboswitch	258548	258760	*gltX*	*cysE*	4.2
Tbox riboswitch	2668833	2669082	*lmo2586*	*lmo2587*	5.4
yybP-ykoY riboswitch	1021463	1021588	*lmo0990*	*lmo0991*	15.6
**ncRNA with lower transcript levels at 20 minutes**					
Rli31	597812	597926	*lmo0558*	*lmo0559*	-3.0
Riboswitch prfA	204339	204468	*prfA*	*plcA*	-3.2
Rli50 - Rli112	2783098	2783274	*lmo2709*	*lmo2710*	-4.1
sRNA ssRS/6S	1546343	1546531	*lmo1513*	*lmo1514*	-4.3
RliG	2386715	2386992	*lmo2302*	*lmo2303*	-4.3
sRNA LhrA	2346166	2346434	*lmo2257*		-5.3
Rli47	2226024	2226532	*lmo2141*	*lmo2142*	-5.6
**ncRNA with higher transcript levels at 24 hours**					
None					
**ncRNA with lower transcript levels at 24 hours**					
Rli60	2054124	2054308	*lmo1982*	*ilvD*	-3.2
Tbox	1642603	1642811	*tyrS*	*ccpA*	-3.2
preQ1	907926	907973	*lmo0866*	*lmo0867*	-3.3
glycine	1372840	1372931	*comGA*	*gcvT*	-3.3
sRNA LhrC2	232086	232197	*cysK*	*sul*	-3.6
sRNA LhrC1	231884	231994	*cysK*	*sul*	-3.6
Unknown	802948	803031			-3.7
Rli125	1154309	1154671	*lmo1117*	*lmo1118*	-3.9
Tbox	1809817	1810057	*lmo1740*	*lmo1741*	-3.9
glmS	756458	756652	*lmo0726*		-4.1
sRNA LhrC3	232289	232400	*cysK*	*sul*	-4.4
SAM	637826	637926	*lmo0595*	*lmo0596*	-4.4
Riboswitch pyrR	1918154	1918262	*pyrR*	*lmo1841*	-4.4
Rli59	1702373	1702553	*lmo1652*	*lmo1653*	-4.5
SAM	1716543	1716651	*metK*	*lmo1665*	-4.6
Rli45	2154765	2154308	*lmo2074*	*lmo2075*	-4.6
Tbox	1588901	1589127	*valS*	*hemL*	-4.7
Rli78	507450	507643	*lmo0470*	*lmo0471*	-4.9
sRNA LhrC4	232492	232605	*cysK*	*sul*	-4.9
pyrR	1917471	1917574	*pyrP*	*pyrR*	-4.9
Rli46	2154775	2154852	*lmo2074*	*lmo2075*	-4.9
Tbox	1597218	1597448	*thrS*	*dnaI*	-5.6
Rli27	434831	434929	*lmo0411*	*lmo0412*	-5.8
Rli37	907576	907832	*lmo0866*	*lmo0867*	-6.1
MboxykoK	2765940	2766104	*lmo2689*	*lmo2690*	-6.8
RliB	544357	544716	*prs*	*lmo0510*	-7.0
Rli53	955829	956001	*lmo0918*	*lmo0919*	-7.7
Tbox	1553972	1554200	*hisS*	*lmo1521*	-8.0
Tbox	2287226	2287453	*lmo2197*	*trpS*	-8.3
SAM	1739491	1739597	*lmo1681*	*lmo1682*	-17.7
Tbox	1676666	1676907	*trpE*	*lmo1634*	-24.7
Rli138	1152549	1152917	*lmo2231*	*lmo2232*	-32.7
**ncRNA with higher transcript level at 20 minutes and 24 hours**					
None					
**ncRNA with lower transcript level at 20 minutes and 24 hours**					
FMN riboswitch	2020487	2020609	*lmo1945*	*lmo1946*	-4.2
Rli61	2275258	2275363	*lmo2187*	*lmo2188*	-4.3

Table S4 (continued). Location, flanking genes, and fold change in expression of the ncRNAs found to be differentially transcribed in the soil extract.

**ncRNA with lower transcript level at 20 minutes and 24 hours**					
Rli48	2361274	2361423	*lmo2271*	*lmo2272*	-4.9
Rli44	2039087	2039375	*lmo1964*	*lmo1965*	-5.3
Unknown	2106073	2106292			-6.6
RliI	2841962	2842200	*lmo2760*	*lmo2761*	-7.6
Rli33-2	708326	708860	*lmo0671*	*lmo0672*	-8.6
