# Supplementary material for: Transcriptomic Analysis of the Adaptation of Listeria monocytogenes to Lagoon and Soil Matrices Associated with a Piggery Environment: Comparison of Expression Profiles
Source: Front Microbiol. 2017 Sep 26;8:1811. doi: 10.3389/fmicb.2017.01811 (PMC5623016; doi:10.3389/fmicb.2017.01811)
Supplement: Supplementary file 5 [file Image1.PDF]

Transcriptomic analysis of the adaptation of *Listeria monocytogenes* to lagoon and soil matrices associated with a piggery environment: comparison of expression profiles

Anne-Laure Vivant[1,2], Jeremy Desneux[1,2], Anne-Marie Pourcher[1,2] and Pascal Piveteau[3] *
[1]Irstea, UR OPAALE, 17 Avenue de Cucillé-CS 64427, F-35044 Rennes, France
[2]Univ Bretagne Loire, France.
[3]Agroécologie, AgroSup Dijon, INRA, Univ. Bourgogne Franche-Comté, F-21000 Dijon,

Figure S1.
Biosynthesis pathways of amino acids for which genes were differentially transcribed in the lagoon effluent.

Caspi R., Billington R., Ferrer L., Foerster H., Fulcher C.A., Keseler I.M., Kothari A., Krummenacker M., Latendresse M., Mueller L.A., Ong Q., Paley S., Subhraveti P., Weaver D.S., Karp P.D.

The MetaCyc Database of metabolic pathways and enzymes and the BioCyc collection of Pathway/Genome Databases

Nucleic Acids Research 44(1):D471-80.(2015)

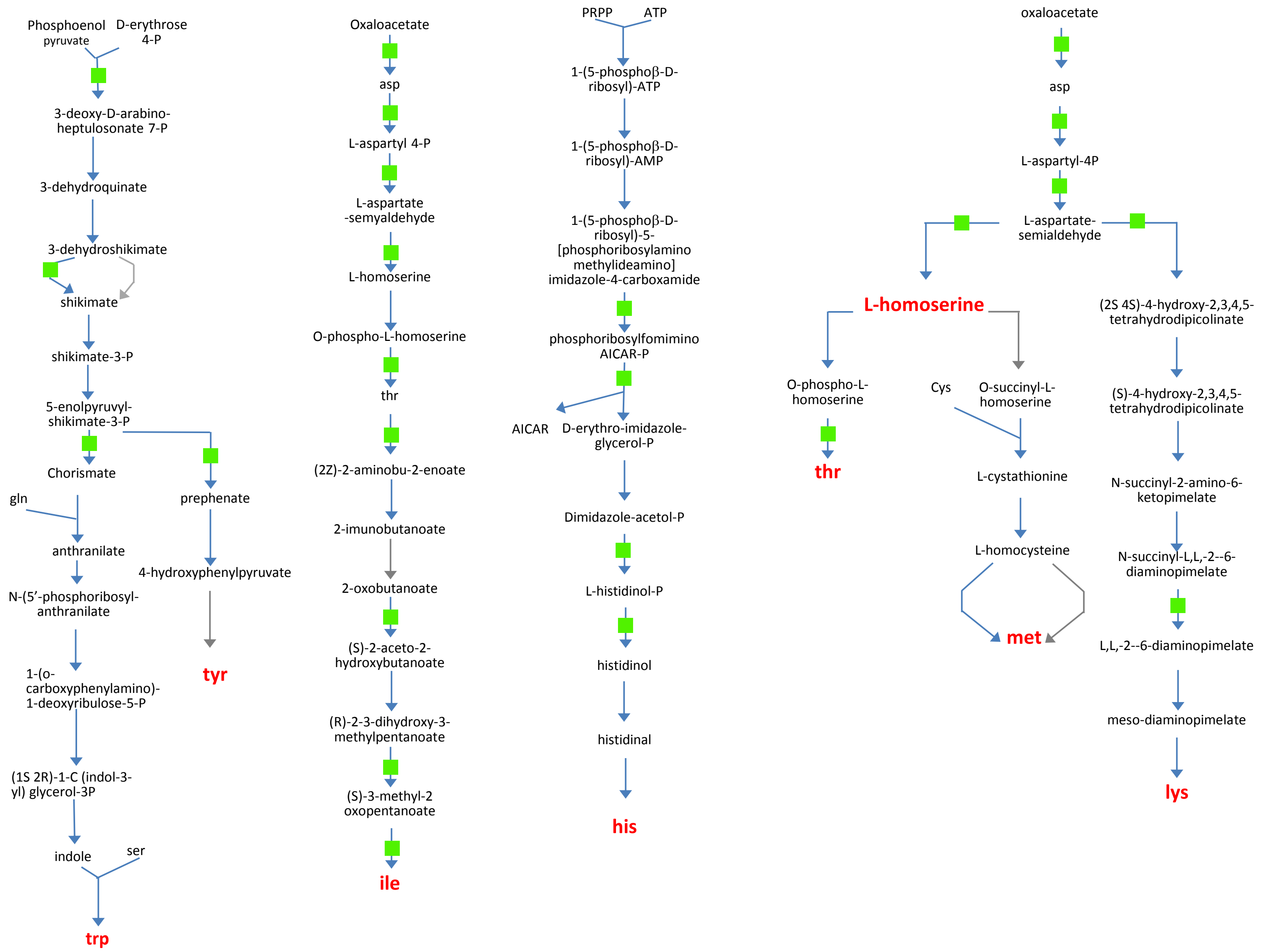

Figure S1. Biosynthesis pathways of amino acids for which genes were differentially transcribed in the lagoon effluent.
Green squares represent open reading frames with a different transcript levels after the incubation of *Listeria monocytog*enes in the lagoon environment

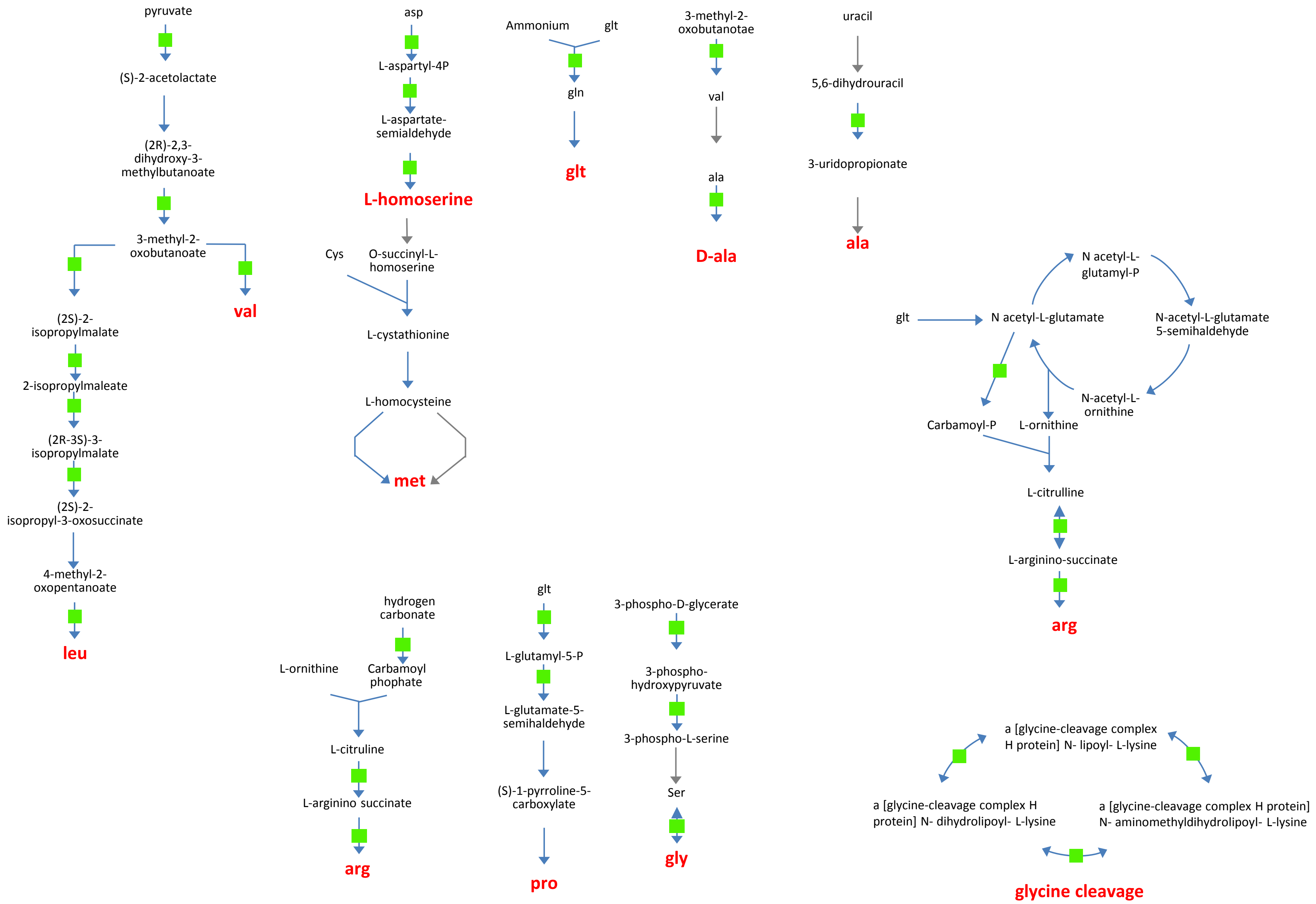

Figure S1 (continued) . Biosynthesis pathways of amino acids for which genes were differentially transcribed in the lagoon effluent.
Green squares represent open reading frames with a different transcript levels after the incubation of *Listeria monocyto*genes in the lagoon environment
