# Supplementary material for: Transcriptomic Analysis of the Adaptation of Listeria monocytogenes to Lagoon and Soil Matrices Associated with a Piggery Environment: Comparison of Expression Profiles
Source: Front Microbiol. 2017 Sep 26;8:1811. doi: 10.3389/fmicb.2017.01811 (PMC5623016; doi:10.3389/fmicb.2017.01811)
Supplement: Supplementary file 6 [file Image2.PDF]

Transcriptomic analysis of the adaptation of Listeria monocytogenes to lagoon and soil matrices associated with a piggery environment: comparison of expression profiles

Anne-Laure Vivant[1,2], Jeremy Desneux[1,2], Anne-Marie Pourcher[1,2] and Pascal Piveteau[3 *]
[1]Irstea, UR OPAALE, 17 Avenue de Cucillé-CS 64427, F-35044 Rennes, France
[2]Univ Bretagne Loire, France.
[3]Agroécologie, AgroSup Dijon, INRA, Univ. Bourgogne Franche-Comté, F-21000 Dijon,

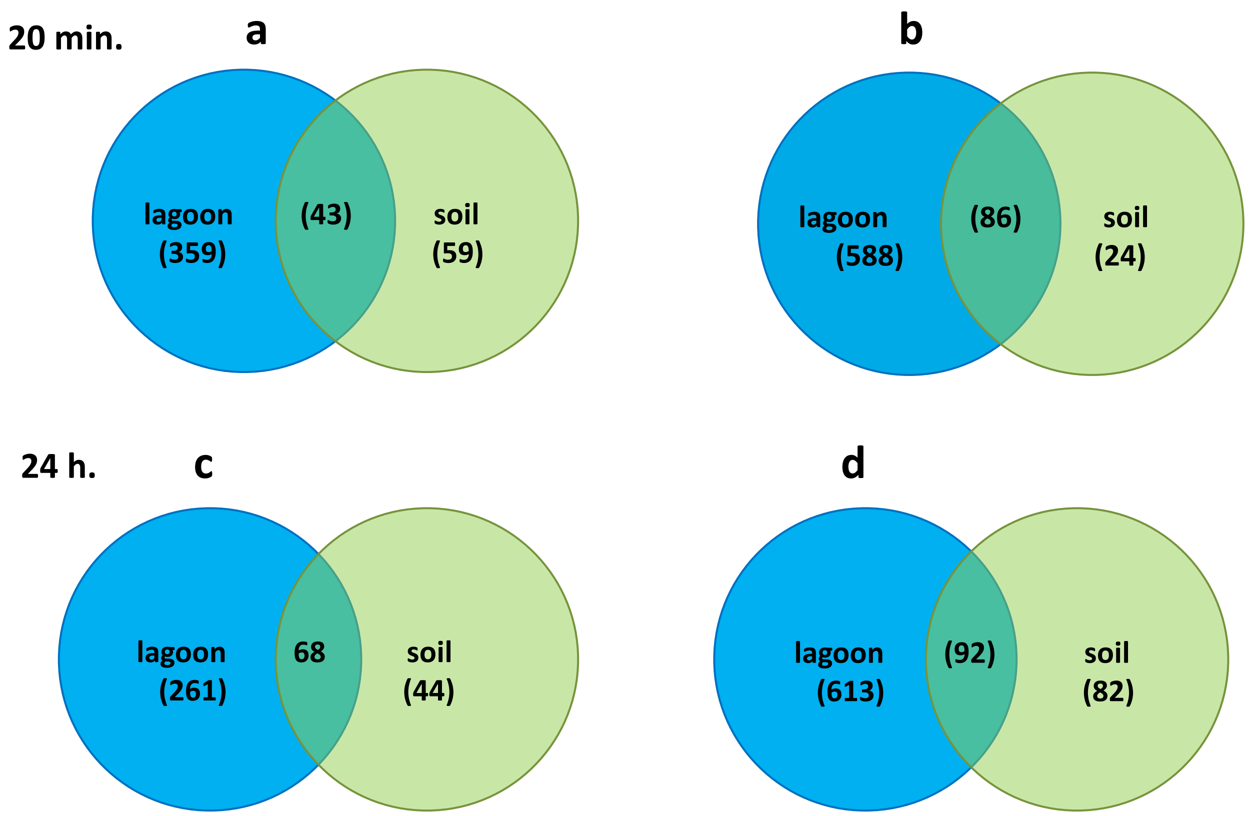

Figure S2. Venn diagrams of genes showing significant differences in soil and lagoon effluent. a) genes with higher transcript levels at 20min. , b) genes with lower transcript levels at 20 min., c) genes with higher transcript levels at 24h. , d) genes with lower transcript levels at 24h.
